# The role of *AtPP2-A3* and *AtPP2-A8* genes encoding Nictaba-related lectin domains in the defense response of *Arabidopsis thaliana* to *Heterodera schachtii*

**DOI:** 10.1007/s00425-023-04196-y

**Published:** 2023-07-08

**Authors:** Kamila Wojszko, Elżbieta Różańska, Mirosław Sobczak, Karol Kuczerski, Tomasz Krępski, Anita Wiśniewska

**Affiliations:** 1 Department of Plant Physiology, Institute of Biology, Warsaw University of Life Sciences, SGGW, Nowoursynowska 159, 02-776 Warsaw, Poland; 2 Department of Botany, Institute of Biology, Warsaw University of Life Sciences, SGGW, Nowoursynowska 159, 02-776 Warsaw, Poland

**Keywords:** Agglutinin, Beet cyst nematode, Plant-parasitic nematode, Plant–nematode interaction, Phloem protein (P-protein), Resistance, Susceptibility, Syncytium

## Abstract

**Main conclusion:**

Expression levels of *AtPP2-A3* and *AtPP2-A8* are reduced in syncytia induced by *Heterodera schachtii* and decline of their expression levels decreases host susceptibility, whereas their overexpression promotes susceptibility to parasite*.*

**Abstract:**

Plant-parasitic nematodes cause huge crop losses worldwide. *Heterodera schachtii* is a sedentary cyst-forming nematode that induces a feeding site called a syncytium via the delivery of secreted chemical substances (effectors) to host cells, which modulate host genes expression and phytohormone regulation patterns. Genes encoding the Nictaba-related lectin domain have been found among the plant genes with downregulated expression during the development of syncytia induced by *H. schachtii* in *Arabidopsis thaliana* roots. To investigate the role of two selected Nictaba-related genes in the plant response to beet cyst nematode parasitism, mutants and plants overexpressing *AtPP2-A3* or *AtPP2-A8* were infected, and promoter activity and protein localization were analyzed. In wild-type plants, *AtPP2-A3* and *AtPP2-A8* were expressed only in roots, especially in the cortex and rhizodermis. After nematode infection, their expression was switched off in regions surrounding a developing syncytium. Astonishingly, plants overexpressing *AtPP2-A3* or *AtPP2-A8* were more susceptible to nematode infection than wild-type plants, whereas mutants were less susceptible. Based on these results and changes in *AtPP2-A3* and *AtPP2-A8* expression patterns after treatments with different stress phytohormones, we postulate that the *AtPP2-A3* and *AtPP2-A8* genes play important roles in the defense response to beet cyst nematode infection.

**Supplementary Information:**

The online version contains supplementary material available at 10.1007/s00425-023-04196-y.

## Introduction

Plant-parasitic nematodes are widespread root pests that cause high economic losses in crops (Jones et al. 2013). The most detrimental sedentary plant-parasitic nematodes belong to the groups of so-called cyst-forming and root-knot nematodes. *Heterodera schachtii*, also known as beet cyst nematode, is a representative of the former. Its infective juveniles (J2 stage) remain dormant inside egg shells stored in protective cysts. After hatching, they are attracted to host roots during migration through the soil. After entering rhizodermal cells, they migrate intracellularly toward the vascular cylinder, where they search for initial syncytial cells, which give rise to a specialized feeding site called a syncytium. The J2s pierce plant cell walls with a stylet and inject secretions produced in the nematode’s two subventral and one dorsal esophageal gland cells. These secretions are thought to be causative agents modifying plant cell metabolism and modulating changes in gene expression to facilitate development of the syncytium that sustains the nematode throughout its entire life cycle. Mature female cyst-forming nematodes are fertilized by vermiform mobile mature males; after oviposition, the females die, and their bodies become protective and highly resistant cysts (Sijmons et al. 1991). The syncytium develops through the formation of partial cell wall dissolutions followed by the fusion of protoplasts of adjacent hypertrophied cells of the vascular cylinder (Golinowski et al. 1996). In the roots of susceptible plants, fully developed and highly efficient syncytia are formed, allowing the development of mature female or male nematodes. In the roots of resistant plants, the syncytia are smaller and apparently less efficient. They usually degrade [due to induction of the hypersensitive response (HR)] or become surrounded by degraded neighboring cells, leading to degradation of the syncytium itself (Sobczak et al. 2005; Varypatakis et al. 2020). These syncytia are usually too short to support development of the females of the nematode, though mature males can develop.

Proteins that comprise the first line of defense of the plant immune system are extracellular surface pattern-recognition receptors (PRRs), containing among others leucine-rich repeat (LRR) domains, which recognize pathogens through activation by pathogen-associated molecular patterns (PAMPs). The proteins secreted by plant nematodes and products of cell wall degradation released during nematode migration may also be recognized as PAMPs. Activation of PRRs leads to a defense response called pattern-triggered immunity (PTI), which inhibits the proliferation of pathogens and disease spread. This phenomenon is referred to as basal resistance (Nürnberger and Lipka 2005). This kind of defense can be interrupted by effectors secreted by successful virulent pathogen isolates in plant cells (Dangl et al. 2013). Thus, the plant becomes susceptible, and the PAMP-induced defense is insufficient to suppress infection and colonization by pathogens. To counteract the action of pathogen effectors, plants have developed resistance (*R*) genes encoding members of the polymorphic superfamily of intracellular NBS-LRR receptors. NBS-LRR proteins possess characteristic domains such as NBS-LRR (nucleotide-binding site-leucine-rich repeats), NB-ARC (nucleotide-binding adaptor shared by Apaf-1*,* R proteins, and CED*-*4) and highly variable domains at their N-terminus, such as TIR (Toll-like/interleukin receptor), CC (coiled coil), RPW8 (resistance to powdery mildew 8), AIG1-type G (avrRpt2-induced), and other domains (Reuber and Ausubel 1996; Arya and Acharya 2018). Particular NBS-LRR proteins are activated by specific pathogen effectors via direct interaction or indirect activation when effectors modify host cellular targets, which then activate NBS-LRR proteins (Dangl et al. 2013). Activation of NBS-LRRs induces a defense response called effector-triggered immunity (ETI), which limits pathogen proliferation (Dangl et al. 2013). In the case of a few examined nematode resistance genes, it seems that NBS-LRRs do not act alone but employ a helper protein, another NBS-LRR. An example of helper proteins found in Solanaceae plants is HR-associated cell death (NRC) proteins. This type of cooperation has been confirmed for tomato Mi-1.2, an R protein, which depends on the presence of NRC4, and for potato Gpa2, an R protein depending upon NRC2 and NRC3 (reviewed by Goverse and Mitchum 2022). To date, only a few nematode *R* genes have been identified in plants and cloned. These genes, mostly originating from wild relatives of crops, have been introgressed into crop plant genomes by crossbreeding, providing useable cultivars. However, under natural field conditions, the resistance can often be overcome by selected virulent/resistance-breaking nematode populations that develop new (sets of) effectors. In *A. thaliana* (ecotype Columbia), the genomic sequences of 149 genes encoding NBS-LRRs have been identified (Meyers et al. 2003). According to Szakasits et al. (2009), the majority of *A. thaliana* NBS-LRR or disease-related genes are downregulated in syncytia at 5 and 15 days post-infection (dpi) with *H. schachtii*. This suggests that *H. schachtii* suppresses the defense responses of *A. thaliana*.

One of the most intriguing research topics in plant immunity is proteins that contain domains typical for plant immunity proteins but are possibly not R proteins themselves. N-terminal motifs characteristic of NBS-LRR proteins can also be found, among others, in members of the PP2-like protein family. By exploring the results of the syncytial transcriptome analysis performed by Szakasits et al. (2009), we found that *PP2-like* genes are strongly up- or downregulated during *H. schachtii* infestation of *A. thaliana* roots (described herein). The PP2-like protein family belongs to the larger lectin family and shares homology with *Nicotiana tabacum* L. agglutinin (abbreviated as Nictaba) (Eggermont et al. 2017). In total, 217 putative genes with lectin domains have been identified in the *A. thaliana* genome. They belong to 9 of 12 different lectin families (Eggermont et al. 2017). Analysis of domains in lectin proteins revealed that they contain at least one lectin domain, which can bind reversibly to specific carbohydrate motifs, free carbohydrates, and glycans from glycoproteins and glycolipids. The lectin domain can be linked to other domains, which have also been identified in proteins playing a role in stress signaling and plant defense (Eggermont et al. 2017). In the *Arabidopsis* genome, 30 orthologs of Nictaba (PP2-like) proteins have been identified, though none contain a signal peptide or transmembrane region (Eggermont et al. 2017). The majority of AtPP2-like/Nictaba proteins possess an F-box domain, four (including AtPP2-A8) orthologs contain an N-terminal Toll/interleukin-1 receptor (TIR) domain, and only one putative Nictaba lectin (AtPP2-A3) possesses an AIG1-type G domain (Eggermont et al. 2017).

Based on structural features and domains of the proteins, as well as on changes in gene expression in syncytia induced by the beet cyst nematode in *Arabidopsis* roots (Szakasits et al. 2009), we selected two *Arabidopsis PP2-like* genes (*AtPP2-A8* and *AtPP2-A3*) for detailed analyses. The AtPP2-A8 protein possesses a Toll/Il-1R (TIR) domain with significant similarity to the TIR domain of the N resistance protein from *Nicotiana glutinosa*, conferring resistance to tobacco mosaic virus (TMV) via pathogen recognition and activation of defense responses (Whitham et al. 1994; Burch-Smith and Dinesh-Kumar 2007). In plants, the TIR domain is involved in the initial interaction with specific ligands that activate intracellular signaling cascades in response to pathogens (Van Der Biezen and Jones 1998). The AtPP2-A3 protein shows high similarity to the defense protein AIG1, which possesses a G domain (AIG1-type G domain) also found in GTPases (Reuber and Ausubel 1996).

The detailed role of PP2-like proteins is not sufficiently understood, similar to their hypothetical involvement in the plant response to soil-borne root pathogenic nematode attack. Therefore, the aim of this work was to provide a detailed functional analysis of two *PP2-like* genes (*AtPP2-A3* and *AtPP2-A8*) in the *Arabidopsis* response to cyst nematode infestation.

## Materials and methods

### Plant materials and culture conditions

The wild-type (WT) *Arabidopsis thaliana* plants ecotype Columbia (Col-0) were used as control in all experiments. *AtPP2-A3* overexpressing transgenic lines (*PP2-A3oe7/1*, *PP2-A3oe9/5* and *PP2-A3oe10/7*), and *AtPP2-A8* overexpressing lines (*PP2-A8oe2/5*, *PP2-A8oe5/3* and *PP2-A8oe8/2*), and mutants (*pp2-a3-1*, *pp2-a3-2* and *pp2-a8-1*) used in this study were also in Col-0 background. Seeds of the T-DNA *pp2-a3* and *pp2-a8* insertional mutants [SALK_045411 and SALK_027300 for *AtPP2-A3* (AT2G26820) and SALK_074249 for *AtPP2-A8* (AT5G45070)] were obtained from the Nottingham Arabidopsis Stock Centre (UK). Seeds were sterilized and plants were cultured in vitro on 0.2KNOP or 0.5MS media under conditions described by Wiśniewska et al. (2022).

### Gene construct preparation and *A. thaliana* transformation

Complementary DNA isolated from segments of uninfected roots of 14-day-old *A*. *thaliana* plants was used to amplify 1584-bp-long coding sequence of *AtPP2-A3* with UTR3’ fragment (NM_001336092.1 splicing form) with the forward primer CACC/ ATGTCAGAGCCAATCAAAAAC and reverse primer CGTTACCACGAAATTTAGA. Forward primer CACC/ ATGGCTGCTTCTTCTTCTGTGAGA and reverse primer TTACTGCGCTGGACGAATTGCAAAG were used to amplify 1065-bp-long coding sequence of *AtPP2-A8*. The polymerase chain reaction (PCR) products were cloned into the pENTR™/D-TOPO® vector (Thermo Scientific, Waltham, MA, USA) and verified by Sanger sequencing. The coding sequences were subcloned into a pK7WG2D vector (https://gatewayvectors.vib.be, accession date 10/28/2022) using Gateway® LR Clonase^®^ II Enzyme mix (Thermo Scientific). The desired constructs were transferred into *Agrobacterium tumefaciens* EHA105 strain by electroporation (MicroPulser; Bio-Rad, Hercules, CA, USA).

Genomic DNA was isolated from frozen *A. thaliana* Col-0 leaves using the Genomic Mini AX Plant kit (A&A Biotechnology, Gdańsk, Poland). Genomic sequences of *AtPP2-A3* and *AtPP2-A8* genes promoters were amplified using primers pairs: CACC/ TTCCTATCCTTTTCTTTTCTGACTTC and ATCGAAGAGGAGAAAAGAAAGAAC (798 bp), and CACC/ AGGGTGAACGCAAAACCTAC and AAGAGAGCTTTTTCTTTTGAGGT (1217 bp), respectively. The PCR products were cloned into pENTR™/D-TOPO^®^ vector (Thermo Scientific) and sequenced using Sanger method. The promoter fragments were subcloned into pKGWFS7 vector containing the kanamycin resistance gene (*nptII*) and *GFP* and *GUS* reporter genes (https://gatewayvectors.vib.be, accession date 10/28/2022) using Gateway® LR Clonase® II Enzyme mix (Thermo Scientific).

Gene constructs containing *PP2-A3* and *PP2-A8* promoters and coding sequences fused with the gene encoding GFP (*pPP2-A3::PP2-A3-GFP* and *pPP2-A8::PP2-A8-GFP*) were obtained using the In-Fusion HD Clontech kit (Takara, Kusatsu, Japan) according to the manufacturer's instructions using the appropriate primers (Table 1). They were cloned into the pK7FWG,0 vector (https://gatewayvectors.vib.be, accession date 10/28/2022) linearized previously with the *EcoR*V restrictase.

**Table 1:** Sequences of primers (5’-3’) used for construction of transgenic *A. thaliana* lines

Gene	Primer type	Primer sequence
*PP2-A3*	Insert 1, Forward with EcoRV site	TCAAGCTAAGCTCTAGTGATATCTTCCTATCCTTTTCTTTTCTGACTT
	Insert 1, Reverse	GCTCTGACATATCGAAGAGGAGAAAAGAAAGAACG
	Insert 2, Forward	CCTCTTCGATATGTCAGAGCCAATCAAAAACA
	Insert 2, Reverse with EcoRV site	CGCCCTTGCTCACCATTGATATCGCGTTTATAGGACGAATTAAAACACCT
*PP2-A8*	Insert 1, Forward with EcoRV site	TCAAGCTAAGCTCTAGTGATATCAGGGTGAACGCAAAACCT
	Insert 1, Reverse	AAGCAGCCATAAGAGAGCTTTTTCTTTTGAGGT
	Insert 2, Forward	AAGCTCTCTTATGGCTGCTTCTTCTTCTGTGA
	Insert 2, Reverse with EcoRV site	CGCCCTTGCTCACCATTGATATCAACTGCGCTGGACGAATTGCA

Transgenic overexpression lines (*PP2-A3oe* and *PP2-A8oe*) containing coding sequences fused to constitutive *35S* promoter, transgenic reporter lines with promoters of *AtPP2-A3* or *AtPP2-A8* fused with the coding sequence of *GUS* reporter gene (*pPP2-A3::GUS* and *pPP2-A8::GUS*), and protein–GFP fusion lines with native promoters of *PP2s* genes (*pPP2-A3::PP2-A3-GFP* and *pPP2-A8::PP2-A8-GFP*) were obtained by the floral dip transformation method (Clough and Bent 1998). Genotype homozygosity was confirmed using the ratio of kanamycin-resistant to non-resistant T2 plants germinating on kanamycin-containing medium.

### Mutants’ genotyping

The homozygosity of the T-DNA insertion lines was confirmed in the relevant DNA sequence sites using PCR and the mutant site-specific primers (Table 2) with SALK_LBb1.3:ATTTTGCCGATTTCGGAAC under the standard PCR conditions recommended by the SALK collection.

**Table 2:** Primers (5´–3´) used for mutants genotyping

Mutant	Donor number	Insertion site	Primers
*pp2-a3-1*	SALK_045411	5´UTR	F: TTCCTATCCTTTTCTTTTCTGACTTC
			G: ATGGAGCACATACCAGGAGTG
*pp2-a3-2*	SALK_027300	Intron 3	F: GTTTTTGGCCCTTTCTTGTTC
			R: GTGACGATTTGGAAGAAGACG
*pp2-a8-1*	SALK_074249	5´UTR	F: CACATGATGAATTGAACGACG
			R: TTGATGAACACTTGAGGTCCC

### RNA extraction and reverse transcription PCR (RT-PCR)

Samples for RNA extraction were collected from segments of uninfected roots and root segments containing syncytia at 5 and 15 days post-infection (dpi) induced in WT, mutants, overexpression lines, and roots of uninfected WT plants treated with JA, (–)-methyl jasmonate (MeJA), SA, and abscisic acid (ABA) (Wiśniewska et al. 2021). Total RNA was isolated and cDNA was synthesized for expression analyses as described by Wiśniewska et al. (2021). Quantitative real-time PCR (qRT-PCR) was performed using SYBR Green Master Mix (Roche, Basel, Switzerland) and 1 μL cDNA as the template for analysis of gene expression in the uninfected and infected roots. Reactions were performed on a LightCycler 96 (Roche). Primers sets: F: GATTGCACAAGCGGAAGCAA and R: AGGTTCATTTTCACCGCTGC, and F: GCAGACATCGAAATTGCAACGA and R: TTCAGCGTCAGGGTCACTTC were used for expression analyses of *AtPP2-A3* and *AtPP2-A8* genes, respectively. Genes encoding dimethylallyl, adenosine tRNA methylthiotransferase (AT4G33380) (Czechowski et al. 2005) (primer F: TTGAAAATTGGAGTACCGTACCAA and R: TCCCTCGTATACATCTGGCCA), Actin2 (AT3g8780) (primer F: CTTGCACCAAGCAGCATGAA and R: CCCCAGCTTTTTAAGCCTTTGATC), Actin8 (AT1G49240) (primer F: ATGAAGATTAAGGTCGTGGCA and R: TCCGAGTTTGAAGAGGCTAC), and GAPDH (AT1G13440) (primer F: TTGGTGACAACAGGTCAAGCA and R: AAACTTGTCGCTCAATGCAATC) were used as references to calculate changes in genes expression. Relative gene expression was calculated according to method of Graeber et al. (2011).

### Nematode infection assay

Infective second-stage juveniles (J2s) of beet cyst nematode (*Heterodera schachtii*) were obtained and plants were inoculated as described previously (Sijmons et al. 1991; Wiśniewska et al. 2021).

### GUS activity assay

Histochemical detection of GUS activity was performed as described by Wiśniewska et al. (2013). GUS activity was examined in seedlings, leaves, flowers, siliques, non-infected roots, as well as in the roots containing 15 dpi syncytia induced by J2s of *H. schachtii* in roots of different genotypes of *A. thaliana*.

### Confocal laser scanning microscopy

Microscopic examinations of transgenic *A. thaliana* lines containing GFP-PP2 proteins’ fusion under control of native promoters (*pPP2-A3::PP2-A3-GFP* and *pPP2-A8::PP2-A8-GFP*) were performed with a Leica TCS SP5II inverted confocal laser scanning microscope (CLSM) (Leica Microsystems, Wetzlar, Germany) on uninfected and nematode infected roots containing 5 dpi syncytia induced by *H. schachtii*. GFP fluorescence emission was monitored in 499–550-nm beam path after excitation with 488-nm line of an argon ion laser.

### Statistical analyses

The significance of differences between means was tested using Fisher’s multiple range test and one-way analysis of variance (ANOVA). The least significant difference (LSD) was calculated at *P* < 0.05. Assumptions of ANOVA, i.e., homogeneity of variances and normality, were checked using statistical tests, Levene’s test, and Shapiro–Wilk test. The p values of these tests were greater than 0.05, which means that the assumptions were fulfilled. All the analyses were performed using Statgraphics software. Gene expression experiments were performed in triplicate. The nematode infection assay was performed in at least five biological replicates per genotype (*n* > 30).

## Results

### *AtPP2-A3* and *AtPP2-A8* gene expression in infected *Arabidopsis* roots

**Fig. 1:**
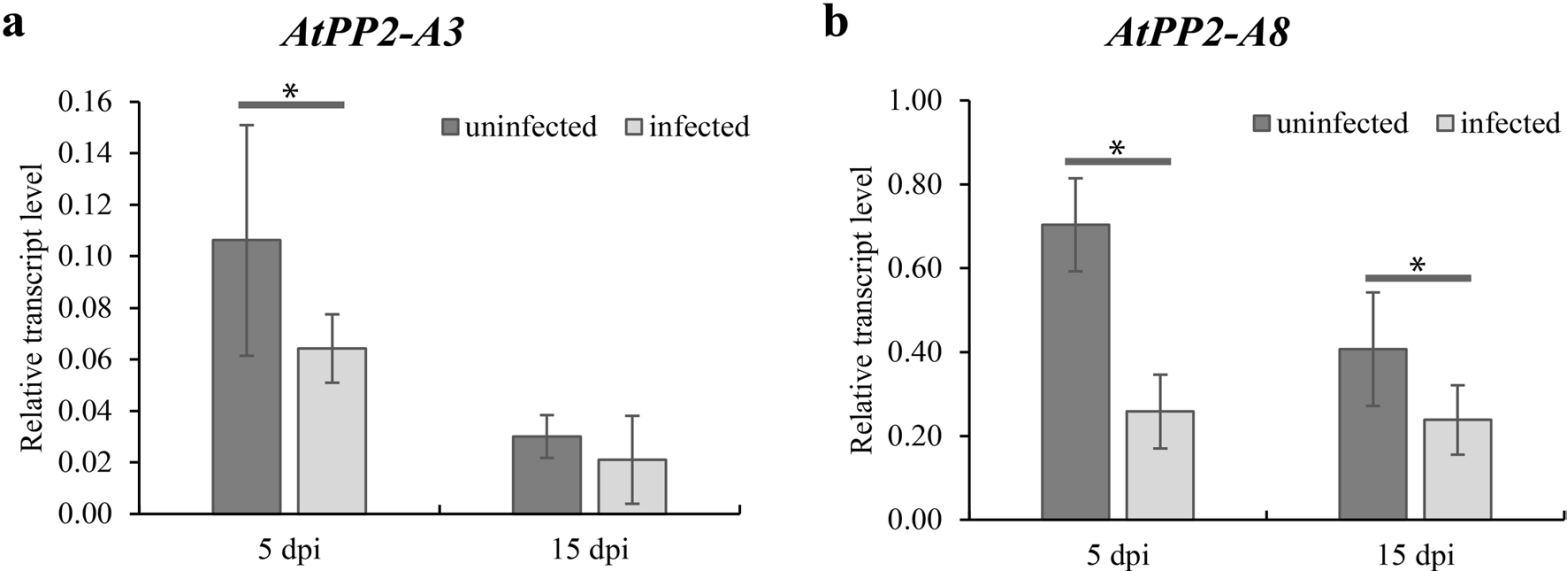
Relative expression levels of *AtPP2-A3* and *AtPP2-A8* genes in syncytia induced by *H. schachtii* in wild-type *A. thaliana*. **a** Relative transcript levels of *AtPP2-A3* in roots containing syncytia at 5 and 15 dpi. **b** Relative transcript levels of *AtPP2-A8* in roots containing syncytia at 5 and 15 dpi. Bars show mean values ± SD. Asterisks indicate means ± SD (*n* = 3), which are significantly different at *P < 0.05 according to one-way ANOVA and post hoc Fisher’s least significant difference (LSD)

*A. thaliana* possesses 30 genes encoding orthologs of Nictaba lectins (PP2*-*like proteins) (Dinant et al. 2003; Eggermont et al. 2017). The analyses of gene expression in syncytia induced by the beet cyst nematode described by Szakasits et al. (2009) showed that two *PP2-like* genes (*AtPP2-B1* and *AtPP2-B11*) were significantly upregulated and four (*AtPP2-A3*, *AtPP2-A8*, *AtPP2-A6*, and *AtPP2-A14*) significantly downregulated. Of the four downregulated genes, we selected two (*AtPP2-A3* and *AtPP2-A8*) with the most decreased transcript accumulation levels (log2 value − 7.2 and − 4.9, respectively) for detailed examination. We used RT-qPCR to confirm the results of Szakasits et al. (2009). The expression level of *AtPP2-A3* in roots containing 5 and 15 dpi syncytia was decreased to 60% (statistically significant) and 69%, respectively, of the expression level in uninfected roots of WT plants (Fig. 1a). The expression level of *AtPP2-A8* was significantly reduced to 36% in infected roots at 5 dpi and 58% in roots with syncytia at 15 dpi in comparison to uninfected roots of WT plants (Fig. 1b). The results of gene expression analysis confirmed downregulation of *AtPP2-A3* and *AtPP2-A8* genes after nematode infection of *A. thaliana* roots.

### Expression of *AtPP2-A3* and *AtPP2-A8* genes in roots treated with phytohormones

**Fig. 2:**
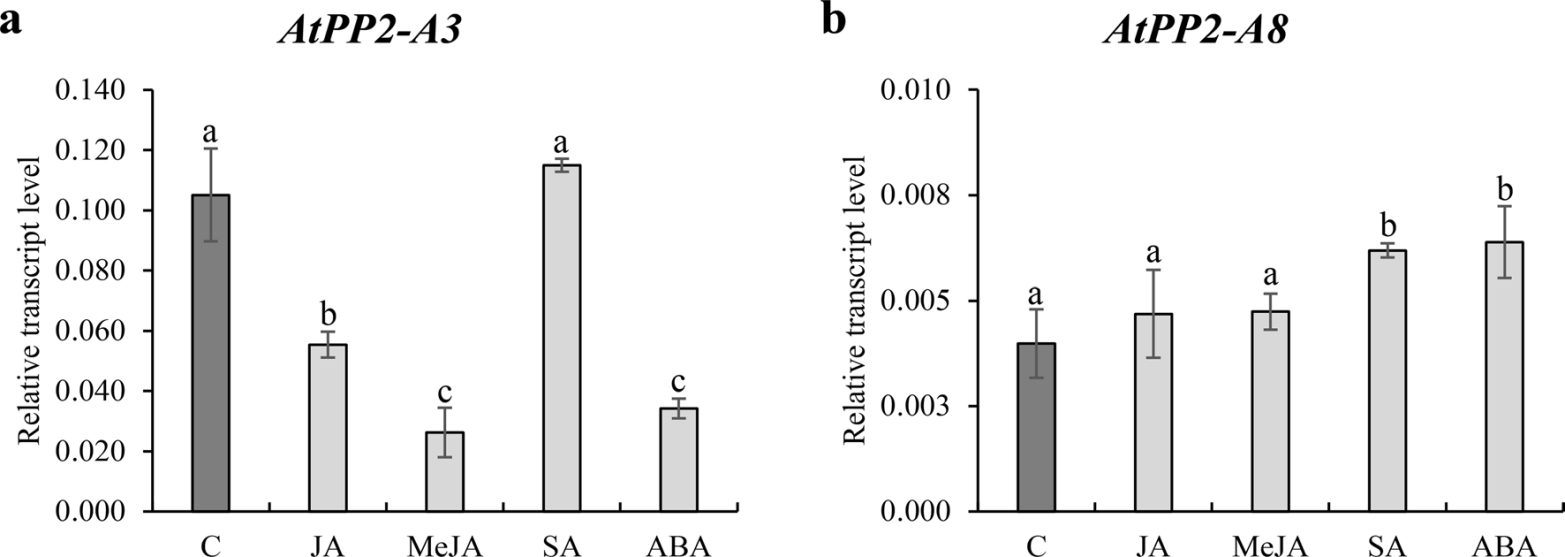
Relative expression levels of *AtPP2-A3* (**a**) and *AtPP2-A8* (**b**) genes in uninfected roots of wild-type *A. thaliana* treated with jasmonic acid (JA), (–)-methyl jasmonate (MeJA), salicylic acid (SA), or abscisic acid (ABA) (24 h treatment with 100 µM concentration each). Bars show mean values ± SD (*n* = 3). Different letters on bars indicate statistically significant differences according to one-way ANOVA (*P* < 0.05) and post hoc Fisher’s least significant difference (LSD)

Expression levels of *AtPP2-A3* and *AtPP2-A8* genes were examined in the uninfected roots of WT plants after 24 h of exposure to jasmonic acid (JA), (-)-methyl jasmonate (MeJA), salicylic acid (SA), or abscisic acid (ABA). The level of *AtPP2-A3* transcript accumulation decreased significantly after treatment with JA, MeJA and ABA in comparison to the water-treated control (Fig. 2a). SA did not influence *AtPP2-A3* expression levels. The level of *AtPP2-A8* transcript accumulation did not change after JA and MeJA treatment but increased significantly after application of SA and ABA (Fig. 2b). Based on the results, we confirmed that JA, MeJA, and ABA downregulated *AtPP2-A3*, but that SA and ABA upregulated *AtPP2-A8*. This difference in expression level changes of both genes after treatment with specific hormones may indicate that their expression is regulated by different mechanisms and regulatory pathways.

### Development of *H. schachtii* on roots of mutant and overexpressing *Arabidopsis* plants

**Fig. 3:**
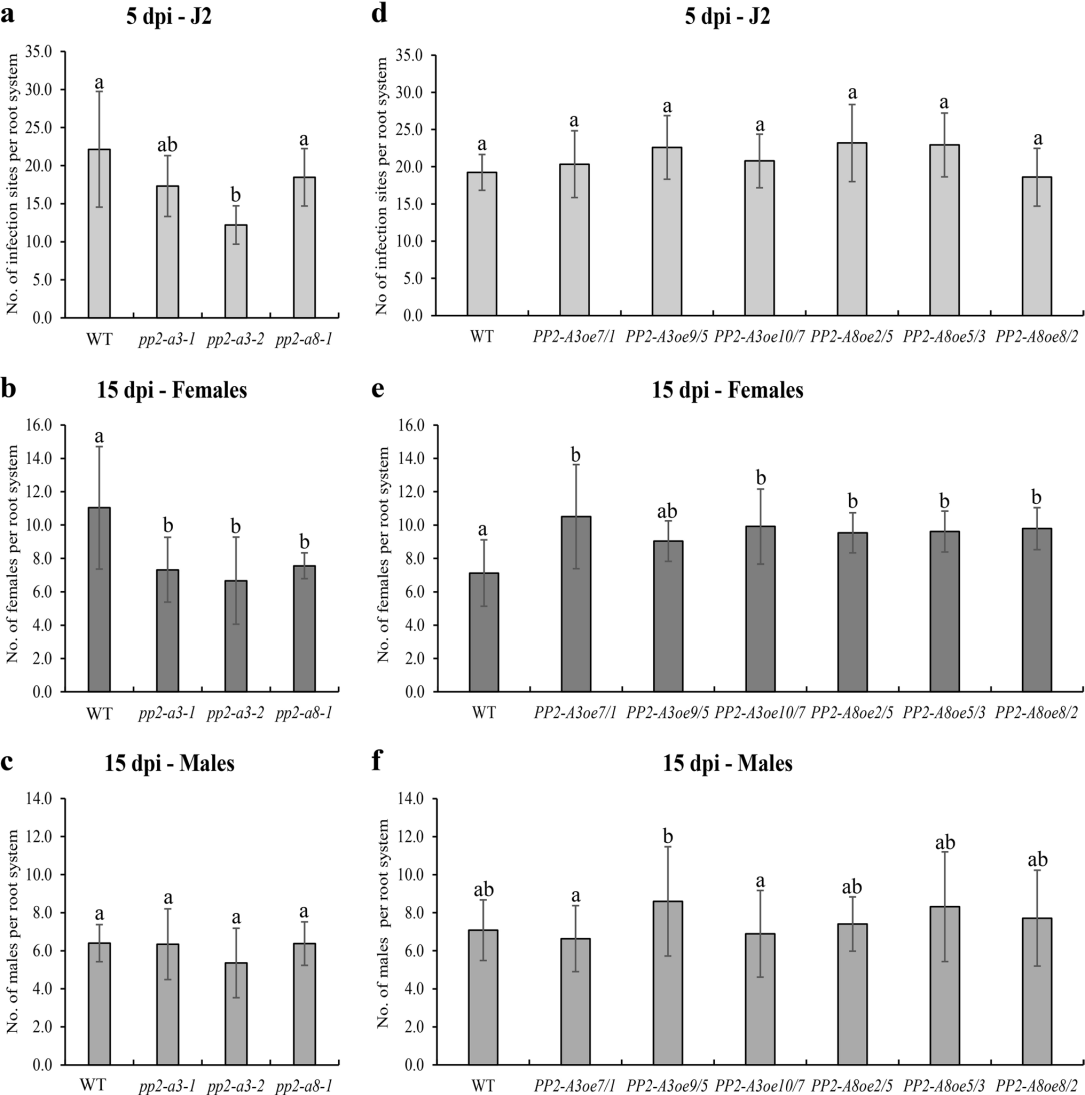
*H. schachtii* development test comparing the susceptibility of wild-type, mutant, and *AtPP2-*overexpressing *A. thaliana* lines at 5 and 15 dpi. **a** Average numbers of sedentary second-stage juveniles developed on mutant roots at 5 dpi. **b** Average numbers of females developed on mutant roots at 15 dpi. **c** Average numbers of males developed on mutant roots at 15 dpi. **d** Average numbers of sedentary second-stage juveniles developed on overexpressing roots at 5 dpi. **e** Average numbers of females developed on overexpressing roots at 15 dpi. **f** Average numbers of males developed on overexpressing roots at 15 dpi. Bars show means ± SD (*n* > 30). Different letters on bars indicate statistically significant differences according to one-way ANOVA (*P* < 0.05) and post hoc Fisher’s least significant difference (LSD)

The potential role of *AtPP2-A3* and *AtPP2-A8* genes in infection of *A. thaliana* roots and development of syncytia induced by beet cyst nematode (*H. schachtii*) was explored using T-DNA mutants (*pp2-a3-1*, *pp2-a3-2*, and *pp2-a8-1*), overexpressing lines (*PP2-A3oe7/1*, *PP2-A3oe9/5*, *PP2-A3oe10/7*, *PP2-A8oe2/5, PP2-A8oe5/3*, and *PP2-A8oe8/2*) and Col-0 wild-type plants. Downregulation of *AtPP2s* expression in mutant roots and upregulation in overexpressing lines were confirmed by RT-PCR (Fig. S1). In the *pp2-a3-2* mutant, significantly lower average numbers of sedentary J2s at 5 dpi (by approximately 45%) (Fig. 3a) and significantly lower average numbers of developed females at 15 dpi for *pp2-a3-1*, *pp2-a3-2*, and *pp2-a8-1* mutants (by approximately 34, 40, and 32%, respectively) (Fig. 3b) per root system were found. Average numbers of males developed on these genotypes at 15 dpi did not differ in comparison to control plants (Fig. 3c). In contrast, the average numbers of females (Fig. 3e) developing on the roots of *AtPP2-A3-* and *AtPP2-A8*-overexpressing lines at 15 dpi were significantly elevated (by approximately 40% for both overexpressed genes) compared to WT plants. However, average numbers of sedentary J2s at 5 dpi (Fig. 3d) and males at 15 dpi (Fig. 3f) showed no significant differences between WT and overexpressing lines. These results suggest that disruption of *AtPP2-A3* and *AtPP2-A8* expression decreases the susceptibility of *A. thaliana* to beet cyst nematode, but that their overexpression elevates susceptibility when expressed as the number of fully developed females. This clearly indicates that proper modulation of *AtPP2-A3* and *AtPP2-A8* gene expression plays a significant role in the response of *A. thaliana* roots to parasitism by *H. schachtii*.

### Expression pattern of *AtPP2-A3* and *AtPP2-A8* genes in *A. thaliana*

**Fig. 4:**
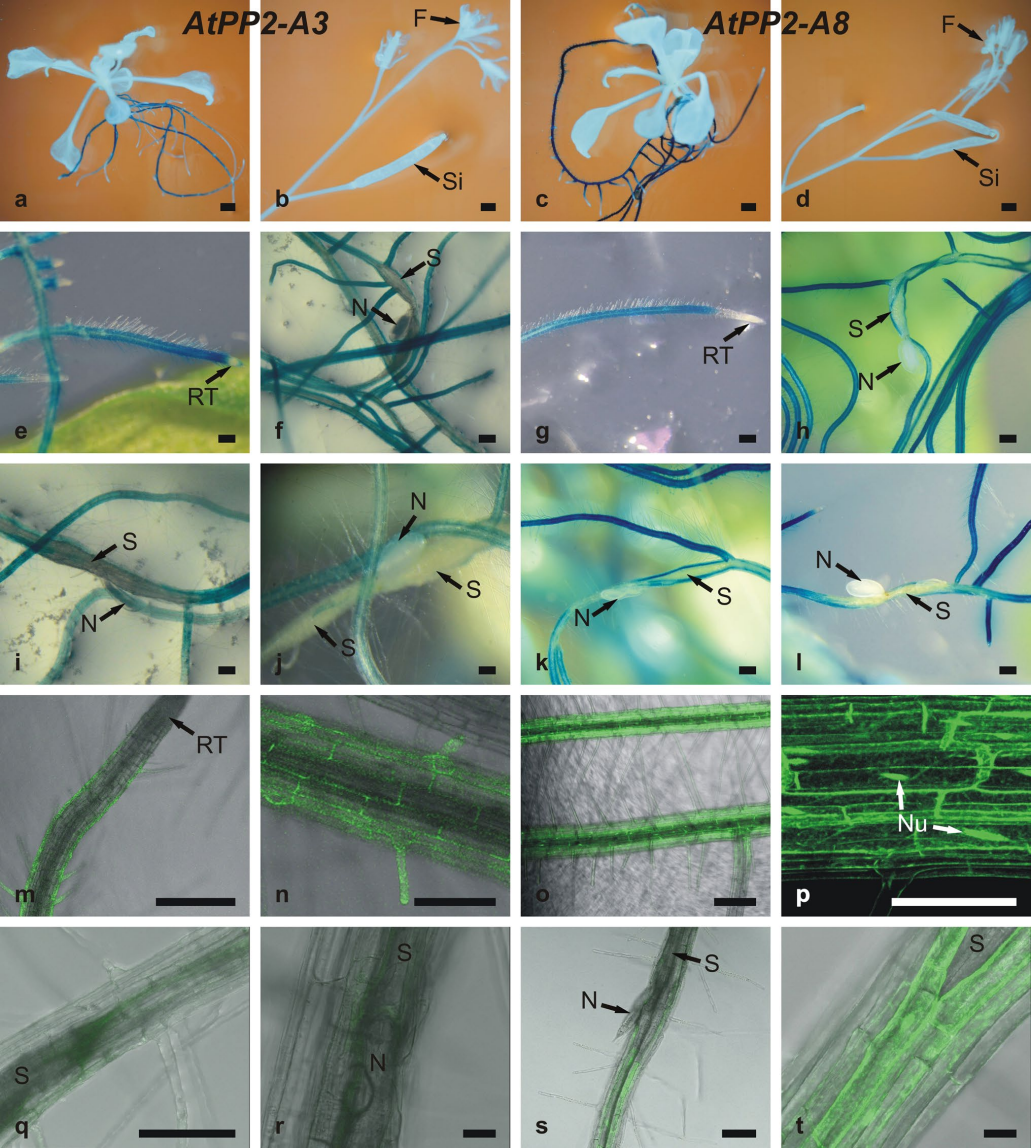
Microscopic analyses of *AtPP2-A3* and *AtPP2-A8* expression in *Arabidopsis* plants. Stereo-light microscope (**a**–**l**) and confocal laser scanning (**m**–**t**) images of transgenic plants expressing the *GUS* reporter gene (bluish coloration) (**a**–**l**) or *GFP* reporter gene (greenish coloration) (**m**-**t**) under control of the *AtPP2-A3* (**a**, **b**, **e**, **f, i**, **j**, **m**, **n**, **q** and **r**) or *AtPP2-A8* (**c**, **d**, **g**, **h**, **k**, **l**, **o**, **p**, **s** and **t**) promoter. **a** and **c** Expression in 12-day-old seedlings. **b** and **d** Expression in inflorescence and siliques. **e**, **g** and **m**–**p** Expression in uninfected roots. **f**, **i**–**l** and **q**–**t** Expression in roots and around syncytia induced by beet cyst nematode. (**f** and **h**–**l** 15 dpi; **r**-**t** 5 dpi). *F* flower, *N* nematode, *Nu* nucleus, *RT* root tip, *S* syncytium, *Si* silique. Scale bars: 1 mm (**a**–**d**); 250 µm (**e**–**h**, **o**); 100 µm (**i**–**n**, **p**, **q**, **s**); 20 µm (**r**, **t**)

Two types of gene constructs for *Arabidopsis* transformation were prepared to determine activities of *AtPP2-A3* and *AtPP2-A8* gene promoters and patterns of their protein localization in uninfected and *H. schachtii*-infected plants. The first type of gene construct contained native promoters linked to a gene encoding β-glucuronidase (*GUS*) (Fig. 4a–l). The second type of construct contained the coding sequence of *AtPP2-A3* or *AtPP2-A8* linked in a single open-reading frame with the *GFP* gene under the control of native *AtPP2-A3* or *AtPP2-A8* promoter fragments (Fig. 4m–t).

Overall, the spatial patterns of *AtPP2-A3* and *AtPP2-A8* gene expression were similar in the organs and tissues of uninfected *A. thaliana* seedlings (Fig. 4a–e and g). In leaves, inflorescence stems, flower buds, flowers, and siliques, the promoters of the *AtPP2-A3* or *AtPP2-A8* genes were inactive (Fig. 4a–d). In uninfected roots, GUS activity was found to be very high, as indicated by the intensity of GUS staining in outer tissues of older fragments of roots (rhizodermis and cortex), except for the apical parts of roots (root tips) and primordia of lateral roots (Fig. 4a, c, e and g). More intense coloration of roots expressing GUS under control of the *AtPP2-A8* gene promoter indicated its higher expression level when compared to the *AtPP2-A3* gene promoter (Fig. 4e, f, i and j versus g, h, k and l).

After root infection with the beet cyst nematode juveniles, the promoter activity of *AtPP2-A3* or *AtPP2-A8* genes indicated by GUS-produced coloration was absent in nematode feeding sites containing nematode-induced syncytia (Fig. 4f and h–l). The results of the GUS activity assay confirmed the previously obtained results of qRT-PCR analyses.

The use of gene constructs in which sequences of *AtPP2-A3* or *AtPP2-A8* genes were fused with the gene encoding the GFP reporter protein under the control of native gene promoters allowed us to obtain more precise information about localization of the proteins (Fig. 4m–t). In uninfected roots, GFP fused with AtPP2-A3 protein was observed mainly in the cytoplasm of root rhizodermis cells (Fig. 4m, n), whereas GFP fused with AtPP2-A8 protein was observed in the cytoplasm and nuclei of rhizodermal and cortical cells (Fig. 4o, p). In roots infected with beet cyst nematode, AtPP2-A3 or AtPP2-A8 protein synthesis was apparently decreased in cells in nematode-induced syncytia and surrounding cells, which, without nematode infection, accumulated the GFP fusion protein (Fig. 4q–t).

Expression pattern analysis of *AtPP2-A3* or *AtPP2-A8* genes revealed expression restricted to outer tissues of roots. Root infestation by *H. schachtii* led to a decrease in expression levels of both genes at infection sites. We also detected *AtPP2-A8* localization in nuclei, which suggests that this gene might play a role in the regulation of transcription.

## Discussion

A protein family consisting of 30 amino acid sequences was identified in the *A. thaliana* genome based on homology to the *N. tabacum* L. agglutinin (Nictaba) family, also known as lectins (Eggermont et al. 2017). The same 30 *Arabidopsis* proteins were previously identified as phloem proteins (P-proteins) based on their homology to CbmPP2 (PP2) from winter squash (*Cucurbita maxima*) and called PP2-like (Phloem Protein 2-like) (Dinant et al. 2003). Unfortunately, no evidence for their synthesis or occurrence in phloem sieve elements or companion cells has been provided thus far. Assigning names to new genes based only on their homology to functionally analyzed genes may result in misinterpretation of the actual role and localization of their putative proteins. The term P-proteins covers all types of specific proteins present in mature and differentiating phloem sieve elements of angiosperm plants (Cronshaw and Esau 1967). Cronshaw and Esau (1967) studied the ultrastructure of *N. tabacum* L. sieve element protoplasts and showed that the P-protein tubules reorganize into shorter striated tubules, which are characteristic of mature sieve elements. Based on this, the tubular P-protein component was designated P1-protein (known currently as Phloem Protein 1, PP1), and the fibrillar component was designated P2-protein (Phloem Protein 2, PP2). Unfortunately, the role and expression patterns of most *Arabidopsis* Nictaba proteins are unclear, and our analyses of AtPP2-A3 and AtPP2-A8 protein localization and their native gene promoter activity do not confirm that they are phloem proteins.

### Expression pattern of *AtPP2-A3* and *AtPP2-A8* genes in *Arabidopsis* roots

The pattern of activity of both tested *AtPP2-A3* and *AtPP2-A8* promoters in *Arabidopsis* tissues was similar, being mainly limited to root tissues, with no signals of activity found in the other organs, such as leaves, flower buds, flowers, or siliques. Detailed analyses using transgenic plants expressing two types of gene constructs showed that promoter activity and synthesis of PP2-A3 and PP2-A8 proteins occurs mainly in the root rhizodermis and cortex but not in phloem cells, as might be supposed by the original names of both genes. These results were astonishing, because the gene promoter activity of two other previously described *Arabidopsis PP2-like* genes (*AtPP2-A1* and *AtPP2-A2*) was detected in vascular tissue (Dinant et al. 2003). Activity of the circa 1 kb-long *AtPP2-A1* gene promoter was detected in the vascular tissue of *Arabidopsis* and in the phloem of the bicollateral vascular bundles of tobacco (*N. tabacum*) stems. A similar pattern of expression, but at lower levels, was observed for the 1 kb-long *AtPP2-A2* gene promoter in transgenic *Arabidopsis*. These results were confirmed by in situ mRNA hybridization experiments. *AtPP2-A1* and *AtPP2-A2* mRNAs were found in companion cell-sieve element complexes, mostly in companion cells (Dinant et al. 2003). However, *AtPP2-B10* gene promoter activity was shown in young leaf trichomes, petioles, major leaf veins, and some mesophyll regions in older leaves (Stefanowicz et al. 2016).

To determine the cellular localization of the products of the *AtPP2-A3* and *AtPP2-A8* genes, C-terminal GFP fusion constructs were used. The AtPP2-A3-GFP and AtPP2-A8-GFP fusion proteins localized to the cytoplasm and nuclei of *Arabidopsis* rhizodermis and root cortex cells. Other Nictaba proteins, i.e., AN4 (AtPP2-A9), AN5 (AtPP2-A1) and AtPP2-A5, have also been found in the cytoplasm and nucleus of *Arabidopsis*, whereas AN3 (AtPP2-A2) is only found in the cytoplasm (Eggermont et al. 2018; Santamaría et al. 2019).

### Differential expression of *AtPP2-A3* and *AtPP2-A8* in response to hormone treatments

Plants use phytohormones to respond to different environmental stresses. The signaling pathways controlled by SA and JA are mainly involved in the response to biotic stress, whereas abiotic stress responses are usually controlled by ABA-related pathways. The role of ABA in response to biotic stress is discussed. To establish feeding sites, plant-parasitic nematodes change plant cell metabolism, among others, by modulating hormonal cross-talk and interactions with hormone pathways (Gheysen and Mitchum 2019).

*AtPP2-A3* and *AtPP2-A8* genes were downregulated after nematode infection and had similar tissue expression patterns, but their expression levels differed after hormone treatments. The expression level of the *AtPP2-A3* gene was downregulated by JA, MeJA and ABA treatment, whereas expression of *AtPP2-A8* was upregulated by SA and ABA.

Expression of other Nictaba genes, *AN3* (*AtPP2-A2*), *AN4* (*AtPP2-A9*), and *AN5* (*AtPP2-A1*), is also differentially regulated (Eggermont et al. 2018). Transcript accumulation of *AN3* was significantly upregulated after treatment with MeJA, ABA, and SA. The expression level of *AN4*(*AtPP2-A9*) was downregulated after MeJA and ABA treatment, and SA treatment practically did not change its expression level. The expression level of *AN5*(*AtPP2-A1*) was slightly upregulated by MeJA treatment and stronger after ABA treatment, and SA treatment practically did not affect its expression (Eggermont et al. 2018).

Sedentary plant-parasitic nematodes need auxin and cytokinin to induce development and to maintain their feeding sites (Gheysen and Mitchum 2019). Stress hormones, such as SA, activate basal defense mechanisms against plant-parasitic nematodes. Although the role of JA in the response to parasitic nematodes is still being discussed, available data indicate that it strongly depends on nematode species, host plant species, and even particular plant genotypes (resistant or susceptible) (Gheysen and Mitchum 2019). Downregulation of *AtPP3-A3* gene expression and upregulation of *AtPP2-A8* by ABA treatment suggest that those genes are also involved in the response to abiotic stresses.

### Role of *AtPP2-A3* and *AtPP2-A8* in defense response to *H. schachtii*

During nematode migration through the rhizodermis and cortex, individual cells within reach of the nematode stylet, including the endodermal cells (the innermost layer of cortex) surrounding the vascular cylinder, become damaged (Holbein et al. 2019). Reaching the vascular cylinder, sedentary cyst-forming plant-parasitic nematodes select a single parenchymatous cell as the initial syncytial cell and inject into it a mixture of effectors that modify host cell metabolism through direct or indirect interaction with host cell proteins. Resistant plants possess PTI or ETI mechanisms to prevent nematode development; susceptible plants trigger inefficient defense pathways to defend themselves (Goverse and Mitchum 2022). To achieve proper syncytium development, nematodes fine tune the expression of different plant genes, and vice versa, plants also modulate expression of their genes participating in the defense response.

Host proteins involved in plant defense responses possess characteristic NBS and LRR domains and N-terminal TIR or CC domains. The *AtPP2-A3* and *AtPP2-A8* genes analyzed herein encode putative lectin domains and domains present in R proteins: AIG1 and TIR, respectively (Burch-Smith and Dinesh-Kumar 2007; Whitham et al. 1994; Reuber and Ausubel 1996). The presence of these characteristic R protein domains in the proteins encoded by the *AtPP2-A3* and *AtPP2-A8* genes, the reduction in their expression levels during nematode attack, and the alteration of gene expression affecting the susceptibility of plants indicate the involvement of *AtPP2-A3* and *AtPP2-A8* in the plant's immune system.

For comparison, the previously described AtPP2-A1 protein does not possess any protein–protein interaction motifs, such as the AIG1-type G domain, TOLL, or F-Box domains located in the N-terminal regions of some other *Arabidopsis* PP2-like proteins (Dinant et al. 2003). It was also shown that AtPP2-A1 binds to several phloem sap proteins and may play different functions in the trafficking of endogenous proteins and in interactions with phloem-sucking insects (Beneteau et al. 2010). AtPP2-A1 did not reduce the number of pea aphids (*Acyrthosiphon pisum*) or green peach aphids (*Myzus persicae*); however, adding the recombinant PP2-A1 protein to the aphid diet caused a delay in weight gain of the nymphs (Beneteau et al. 2010). Overexpression of the *AtPP2-A1* gene caused repression of *M. persicae* phloem-feeding activities due to molecular interaction between Mp1 (effector aphid salivary protein) and AtPP2-A1 (Wang et al. 2021), which led to reduced insect colonization (Zhang et al. 2011). In contrast, upregulation of *AtPP2-A1* after pathogen attack and antifungal activity of its protein have been described (Lee et al. 2014). Overexpression of the *AtPP2-B10* gene encoding the F-Box domain, expression of which is elevated after plant infection with the virulent *Pseudomonas syringae* pv. *tomato* strain DC3000 (*Pst* DC3000), resulted in reduced leaf damage at infection sites; in contrast to infected mutant and wild-type plants, it also caused a reduction in bacterial numbers and accumulation of anthocyanins (Stefanowicz et al. 2016; Romero-Pérez et al. 2021). Other Nictaba orthologs, such as *AN3* (*AtPP2-A2*), *AN4* (*AtPP2-A9*), and *AN5* (*AtPP2-A1*), of *Arabidopsis* encode only the lectin domain, for which participation in the defense response against bacterial pathogens was confirmed. Overexpression of *AN4*(*AtPP2-A9*) and *AN5*(*AtPP2-A1*) significantly improves tolerance to *P. syringae* compared to wild-type plants (Eggermont et al. 2018). It was also shown that the *AtPP2-A5* gene, encoding a protein containing lectin and TIR1 domains, confers tolerance to herbivorous two-spotted spider mites (*Tetranychus urticae*) through modulation of phytohormonal signaling. Overexpression or mutation of the *PP2-A5* gene results in transcriptional reprogramming that modulates the balance of hormone accumulation and corresponding signaling pathways (Santamaría et al. 2019).

The role and localization of PP2-like protein activity varies depending on the amino acid sequence. In our work, we showed that modification of expression levels of the *AtPP2-A3* and *AtPP2-A8* genes via mutation or overexpression induced divergent responses to attack by cyst-forming nematodes. Mutations in the *AtPP2-A3* and *AtPP2-A8* genes led to a decrease in plant susceptibility to *H. schachtii*, whereas their overexpression had the opposite effect, namely, an increase in the susceptibility of transgenic *Arabidopsis* plants. Moreover, modulating *AtPP2-A3* and *AtPP2-A8* gene expression levels in mutant or overexpressing lines had a greater impact on the numbers of females than males and a relatively weaker effect on juveniles. This result suggests that these genes mainly act at later stages of nematode development and are putatively nematode sex dependent. Differential gene expression depending on whether it is a female- or male-associated syncytium has been demonstrated previously (Anjam et al. 2020).

*AtPP2-A3* and *AtPP2-A8* are unique genes that are downregulated by the host during nematode infestation to decrease plant susceptibility. Most likely, these genes act as negative regulators of defense pathways under regular conditions and are downregulated after pathogen attack in *Arabidopsis* roots to switch on the plant defense response. Sedentary plant-parasitic nematodes are able to manipulate the metabolism and gene expression of host plants to develop a specialized feeding structure, resulting in crop yield reduction. One of the solutions to this agricultural problem is plant resistance breeding. Unfortunately, the number of known and available *R* genes is limited. Therefore, for over 2 decades, scientists have focused on identifying and analyzing susceptibility (*S*) genes. *S* genes can be modified by genome editing tools and be used to obtain plants with increased tolerance or resistance. Unfortunately, *S* genes often show a pleiotropic effect, and their damage can negatively affect important physiological processes. Therefore, research in the field of functional genomics, to which we can add our work, increases the possibility of collecting gene pools to gain double or triple mutants that can be used to obtain cultivars with increased tolerance to nematodes. However, further investigation of the *AtPP2-A3* and *AtPP2-A8* genes to explain their function and interactions with other potential components of the plant defense system is required to make progress in understanding their mode of action.

#### Author contribution statement

AW developed the concept and designed the experiments, analyzed the results, performed the statistical analyses, genotyped the mutants, conducted the infection tests, and participated in and coordinated all the molecular analyses. KW and KK performed the mutant and transformant genotyping. KW, KK, and TK prepared the gene constructs and transformed plants. ER and MS performed the infection tests and microscopic analyses. KW performed the gene expression analyses. AW and MS wrote the manuscript. All authors discussed the results and commented on the manuscript.

## Supplementary Information

Below is the link to the electronic supplementary material.

Supplementary file1 (PDF 264 KB)

## Data Availability

The datasets used and/or analyzed during the current study are available from the corresponding author on reasonable request.

## Abbreviations

dpi: Days post-infection
J2: Infective juveniles (J2 stage)
Nictaba: *Nicotiana tabacum* L. agglutinins (lectins)
OE: Overexpressing
PP: Phloem protein
