# Supplementary material for: The role of AtPP2-A3 and AtPP2-A8 genes encoding Nictaba-related lectin domains in the defense response of Arabidopsis thaliana to Heterodera schachtii
Source: Planta. 2023 Jul 8;258(2):40. doi: 10.1007/s00425-023-04196-y (PMC10329053; doi:10.1007/s00425-023-04196-y)
Supplement: Supplementary file 1 — Supplementary file1 (PDF 264 KB) [file 425_2023_4196_MOESM1_ESM.pdf]

Supplementary materials

The role of *AtPP2-A3* and *AtPP2-A8* genes encoding Nictaba-related lectin domains in the defense response of *Arabidopsis thaliana* to *Heterodera schachtii*

Kamila Wojszko[1], Elżbieta Różańska[2], Mirosław Sobczak[2], Karol Kuczerski[1], Tomasz Krępski[1], Anita Wiśniewska[1]*

[1] Department of Plant Physiology, Institute of Biology, Warsaw University of Life Sciences - SGGW, Nowoursynowska 159, 02-776 Warsaw, Poland
[2] Department of Botany, Institute of Biology, Warsaw University of Life Sciences - SGGW, Nowoursynowska 159, 02-776 Warsaw, Poland

Analysis of *AtPP2-A3* and *AtPP2-A8* transcript accumulation

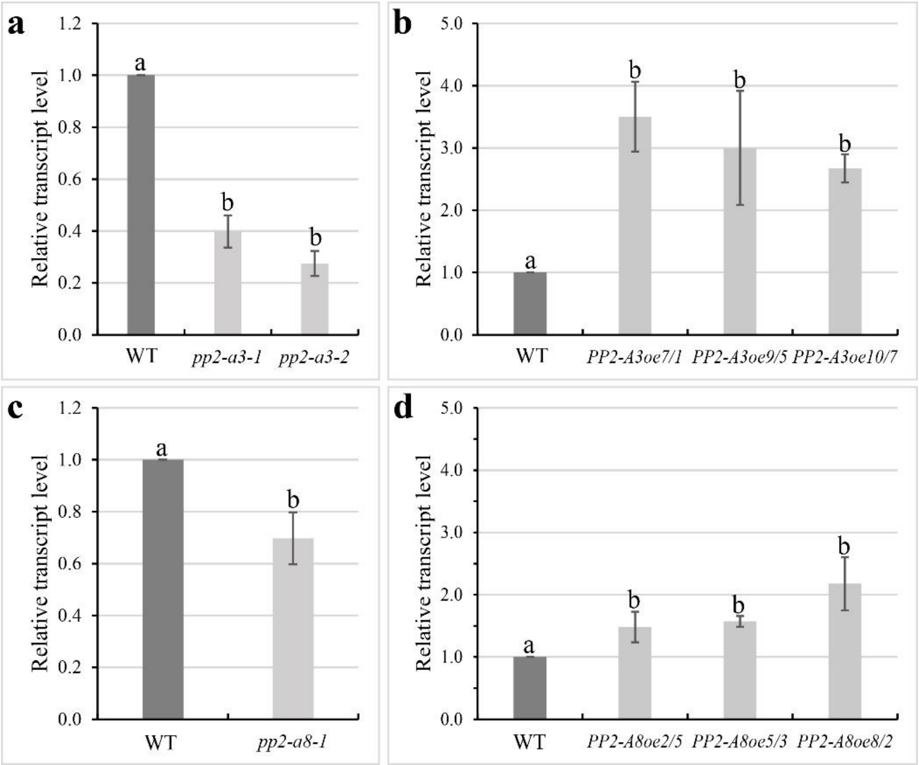

Fig. S1 The relative expression levels of *AtPP2-A3* and *AtPP2-A8* genes in mutant and overexpressing *Arabidopsis thaliana* plants. **a** Relative transcript levels of *AtPP2-A3* gene in mutant plants. **b** Relative transcript levels of *AtPP2-A3* in overexpressing lines. **c** Relative transcript levels of *AtPP2-A8* gene in mutant plant. **d** Relative transcript levels of *AtPP2-A8* in overexpressing lines. The bars show mean values ± standard deviation from 3 biological and 2 technical repeats. Transcript levels were analyzed by RT-PCR and the presented values are relative to those obtained for WT (given the value of 1), after normalization to the level of *Actin2* RNA with primers mentioned in Materials and Methods section. a, b homogenous groups (P <0.05; ANOVA; LSD)
